# Influence of Nutrition Claims on Appetite Sensations according to Sex, Weight Status, and Restrained Eating

**DOI:** 10.1155/2016/9475476

**Published:** 2016-09-20

**Authors:** Geneviève Painchaud Guérard, Simone Lemieux, Éric Doucet, Sonia Pomerleau, Véronique Provencher

**Affiliations:** ^1^Institute of Nutrition and Functional Foods, Université Laval, 2440 Boulevard, Hochelaga, Quebec City, QC, Canada G1V 0A6; ^2^School of Human Kinetics, University of Ottawa, 75 Laurier Ave East, Ottawa, ON, Canada K1N 6N5

## Abstract

Nutrition claims may help people to adopt healthier eating habits, but little is known about the potential cognitive effects of such claims on appetite sensations. The main purpose of this study was to evaluate the impact of nutrition claims and individual factors on perceived appetite sensations. According to a three (“healthy” versus “diet” (i.e., satiating) versus “hedonic”) by two (restrained or not restrained) by two (normal-weight or overweight/obese) by two (men versus women) factorial design, 164 males and 188 females aged 18–65 were invited to taste an oatmeal-raisin snack in a blinded and* ad libitum* context. Visual analog scales (150 mm) were used to evaluate appetite sensations before and over 1 h after consumption period. BMI and Restraint Scale were used to categorize participants according to their weight and restraint status. No main condition effect was observed for any of the four appetite sensations. However, subgroups analysis revealed significant differences among specific subgroups. A main effect of sex was also observed for all appetite sensations with men reporting higher levels of desire to eat, hunger and prospective food consumption, and lower levels of fullness than women. These findings highlight the importance of considering individual characteristics in interaction when studying appetite sensations.

## 1. Introduction

The increasing prevalence of obesity represents a major public health concern in many countries, and healthy eating can play a predominant role in prevention strategies [1]. In this context, it is important to identify effective nutritional strategies to help individuals achieve or maintain a healthy weight by making better food choices. One of these strategies consists in the regulation of food labeling, which includes the use of nutrition claims intended to provide a quick and easy way to identify foods with nutritional features of interest [2–4]. In this regard, satiating properties of foods induced by specific nutrients such as fibres or proteins are of particular interest [5, 6]. Indeed, there is convincing evidence that appetite sensations are important determinants of long-term energy intake [7, 8] and that satiety-enhancing foods may provide benefit to consumers by facilitating appetite control and compliance with weight-management efforts [9]. Furthermore, the regulation of nutrition claims related to satiety is currently evaluated and developed by health organizations, which clearly recommend that such claims are supported by strong scientific evidence [10, 11]. While nutritional information related to the satiating effects of foods might be relevant for weight control, less is known about consumers' perception and understanding of such nutrition claims and how these claims can affect perceived appetite sensations.

Indeed, nutritional information conveyed by food labeling can be difficult to understand for many consumers, particularly for older adults, adolescents, or those with less education [12, 13]. Nutrition claims can also be misunderstood, which may lead to misleading inferences or unexpected eating behaviors. For example, a recent study showed that a low-fat-labeled candy was perceived healthier than a regular-labeled candy, independent of caloric information [14]. It is also recognized that low-fat claims can lead consumers to overconsumption [15]. Similarly, another study showed that a snack described as “healthy” was considered less fattening and that the amount eaten was 35% higher than when the snack was described as “unhealthy” [16].

With regard to appetite sensations, it has been demonstrated that a food considered as unhealthy is perceived to contribute more to weight gain and satiation [17], while imposed healthy eating can increase the sensation of hunger [18]. Beliefs and expectations about the satiating power of a food can also affect perceived fullness, and this effect can persist as long as three hours after the meal [19]. Another study showed that a meal presented as highly satiating, without having physiological satiating properties, can increase satiety potential more than a meal having physiological satiating properties, but presented as regular [20]. This suggests that the psychological effect of a satiety-related claim can be as effective as a physiological effect related to intrinsic satiety properties and highlights the need to better understand this possible psychological effect.

Individual factors such as sex, weight, or restrained eating could also influence the perception of foods and modulate behavioral change. As the perception of food can influence appetite sensations, it could be hypothesized that individual factors can also modulate the perception of appetite sensations. Since women generally consider themselves as being more knowledgeable about nutrition and having better eating habits [21] and tend to pay more attention to nutritional information [22], it could be suggested that women would be more readily influenced by nutrition claims. Overweight/obese individuals and restrained eaters might also be more sensitive to nutrition claims related to healthy eating or weight loss since they can use it as a tool for weight management. Interestingly, one study showed that food products carrying satiety-related claims were generally not seen as a magic bullet to weight loss, but that restrained eaters were more prone to overinterpretation of these claims, that is to interpret it as directly delivering weight loss [23]. Individual factors can thus affect perception of foods and perceived appetite sensations, but they can also act in a physiological way. Indeed, some studies have associated higher levels of restraint with an orexigenic hormonal profile [24, 25], suggesting that restrained individuals could more easily feel hunger. It has also been proposed that overweight/obese individuals are less connected to their internal signals than normal-weight individuals, making the detection of hunger and satiation more complex [26]. Sex, weight, and restrained eating are thus variables of interest when studying appetite sensations, alone or in combination with nutrition claims.

In summary, current literature suggests that nutrition claims may influence perception of foods and appetite sensations and that this influence could be modulated by individual factors such as sex, weight, or restrained eating. In that context, there is an urgent need to better understand the way nutrition claims should be used, taking into account possible side effects, and evaluate the relevance of some claims in a public health nutrition context. This study addresses this issue by evaluating combined and separated effects of nutrition claims and individual factors on appetite sensations.

The main purpose of this study was to evaluate the impact of nutrition claims and individual factors on perceived appetite sensations. The specific objectives were (1) to evaluate the effects of “healthy,” “diet,” or “hedonic” claims on appetite sensations, (2) to evaluate if sex, weight, or restrained eating influence appetite sensations, and (3) to investigate if sex, weight, and restrained eating can modulate the effects of nutrition claims on appetite sensations. Note that the “hedonic” condition refers to ingredients generally considered as highly palatable and that the “diet” condition refers to satiating properties (see Section 2.1 for a complete description). Our corresponding hypotheses are that (1) the snack in the “healthy” condition will be less satiating than in the “diet” or “hedonic” conditions, (2) individual factors such as sex, weight, and restrained eating will significantly influence appetite sensations, and (3) the satiating effects of the “diet” and “hedonic” conditions will be more important in overweight/obese restrained women.

## 2. Material and Methods

### 2.1. Participants and Study Design

The study was conducted among 164 men and 188 women (aged 18–65) from Quebec City, Canada, who were invited to taste and rate an oatmeal-raisin snack in a blinded and* ad libitum* context. Exclusion criteria were personal condition or history of diseases that could affect food intake (unstable weight in the last two months, pregnancy, breastfeeding, eating disorders, type 1 or 2 diabetes, and uncontrolled hypo- or hyperthyroidism), use of medication that might interfere with appetite or food taste (antidepressants, antipsychotic, or corticosteroids), and food allergies for safety reasons. Participants were randomly assigned to an experimental condition in a three (“healthy” versus “diet” versus “hedonic”) by two (restrained or not restrained) by two (normal-weight or overweight/obese) by two (men versus women) factorial design, which means that the recruitment was based on these characteristics in order to obtain a balanced number of participants in each subgroup. This design results in eight subgroups based on individual characteristics combined with the three experimental conditions tested, which leads to a total of 24 combinations for the analysis. The factorial distribution of participants is presented in Table 1.

Initial randomization was performed according to self-reported weight and height and restraint score, but participants were reassigned according to their measured data if needed. The “healthy” condition emphasized the favorable nutritional characteristics of the snack: “The snack product that you have to taste today is a new high-fiber oatmeal snack made with healthy ingredients. You have certainly heard that whole oatmeal is good for your health because it contains soluble fibers. So, this new oatmeal snack is high in soluble fibers, as well as low in saturated fat and free from trans-fat.” The “diet” condition focused more on beneficial satiating properties for weight management: “The food product that you have to taste today is a new healthy high-fiber snack. You must have probably heard that whole-grain foods, like those that contain oat soluble fibers, are composed of complex carbohydrates which are digested slowly and which can delay the appearance of hunger. This new healthy snack has been especially designed to satiate, because it is a source of fibers, which make it an interesting choice for any persons concerned to reach and to maintain a healthy weight.” Finally, the “hedonic” condition underlined the sweet and pleasant taste of the snack using hedonic food words [27]: “(These are) new gourmet cookies made with fresh butter and old-fashioned brown sugar. So, these new cookies are a great treat with a pleasant, sweet taste.” Note that the word “cookie” was never mentioned in the two first conditions, and that we specifically chose to use an oatmeal-raisin rather than a chocolate snack in order to facilitate the blinded context of the experiment, as oatmeal and raisins can be considered as healthy ingredients or not depending on the food containing it. Indeed, this psychological manipulation was previously shown to be effective in changing the healthiness perception of food [16, 28]. In spite of the description, the snack was exactly the same in all conditions. Participants were blinded to the main goal of the study. The study was approved by the institutional review board of* Université Laval* (#2009-117/26-05-2009) and was registered in the Clinical-Trials.gov registry (NCT01141140).

### 2.2. Visit Details

Participants came to the Institute of Nutrition and Functional Foods (Université Laval) between 8:30 a.m. and 7 p.m. at a single occasion. They had to be in a fasting state for at least two hours prior to their visit. They were given a food description according to the experimental condition they were assigned and then had 10 minutes to taste the snack* ad libitum* in a private room. Leftovers were weighed in grams to calculate the amount eaten. Participants stayed for approximately two hours after the taste-rating task to complete appetite sensations scales, questionnaires, and anthropometric measurements. They were told the real goal of the study only at the end of the visit and were free to withdraw their participation at that moment.

### 2.3. Appetite Sensations

Appetite sensations are defined as follows: hunger refers to the physiological recognition of a need to eat, while desire to eat reflects the motivation to eat [29], fullness is a physical feeling that could be related to the degree of stomach filling [30], and satiety refers to the processes that inhibit further intake after an eating occasion has ended [31]. Appetite sensations were evaluated with unipolar 150 mm visual analog scales (VAS) (adapted from Hill and Blundell [32]). These scales are anchored at the two ends with the extremes of the subjective feeling evaluated. VAS have been widely used in the past to evaluate appetite sensations and are considered reliable for single meal protocols [31, 33]. Participants rated their appetite sensations before the test meal (time = −10) as well as immediately after (time = 0) and 20, 40, and 60 minutes later. The first measure (time = −10) was taken immediately before the description of the snack. Four appetite sensations were measured: desire to eat (“How strong is your desire to eat?”; “Very low desire” to “Very high desire”), hunger (“How hungry do you feel?”; “Not at all hungry” to “Extremely hungry”), fullness (“How full do you feel?”; “Not at all full” to “Extremely full”), and prospective food consumption (PFC) (“How much food do you think you could eat?”; “None at all” to “A large amount”). The VAS values were calculated by measuring the distance in mm with a ruler from the left end of the scale to the mark drawn by the participant with a precision of 0.5 mm. One-hour area under the curve (AUC) was calculated from time zero to time 60 with the trapezoid method [34]. As recommended by Blundell et al. [29], total AUC was used rather than the incremental AUC. The AUC allows an overall view of the appetite sensation response, while the raw VAS data allows comparisons at each time.

### 2.4. Questionnaires

Questionnaires were administered after the taste-rating task. Appreciation of the snack (“In general, how much do you appreciate the food you just tasted?”) was evaluated with a unipolar 150 mm VAS labeled from bottom to top with “Not at all,” “Moderately,” and “Enormously.” A validated Food Frequency Questionnaire (FFQ) was administered by a registered dietitian in order to determine usual food intake [35]. Anthropometric data, age, and physical activity level were also collected. The Restraint Scale allowed the distinction between restrained eaters (score ≥ 12 for men and ≥ 15 for women) and unrestrained eaters (score < 12 for men and < 15 for women) [36]. Restrained eating is defined as behavioral and attitudinal concerns about dieting and weight control. The validity of the Restraint Scale for the measurement of restrained eating has been previously reported [37–39]. The Food Pleasure Scale (FPS) was also used to evaluate the pleasure associated with eating [40].

### 2.5. Anthropometric Measurements and Energy Needs

Body mass index (BMI) was calculated from weight and height measured after the completion of all questionnaires. Participants were classified as normal-weight if they had a BMI under or equal to 25 kg/m^2^ and as overweight/obese if they had a BMI higher than 25 kg/m^2^. Energy needs were estimated from the mean of FFQ data and the Harris-Benedict formula [41], as previously performed in our research institute [42].

### 2.6. Statistical Analysis

With a mean Cohen's *d* estimate of 0.35 [43], power analyses for ANOVAs testing main effects and interactions indicated that a sample size of 180 males and 180 females would allow the detection of significant differences with an alpha level of 0.02 and a power (1 − *β* error probability) of 0.90. One-way analyses of variance (ANOVAs) were used to assess differences in initial characteristics between experimental conditions. In line with the factorial design of the study, factorial repeated measures ANOVAs were performed with MIXED procedures for the analysis of the appetite VAS (desire to eat, hunger, fullness, and PFC), with time, experimental condition, sex, BMI, and restrained eating used as independent variables and tested in interactions. Age and initial appetite sensations (time = −10) were included in the models as potential confounding factors, as they may affect appetite sensations [29, 44]. Adjustments for energy needs and total amount eaten were also made to palliate to the fact that the amount eaten was* ad libitum* and therefore not adjusted for energy requirements (as the study also aimed to measure the effect of claims on food intake). Time of the day was also added as a covariate in case the two hours fasting period was not long enough to standardize initial appetite sensations. Finally, appreciation of the snack and pleasure associated with eating were added as covariates as hedonic eating indicators [45]. No multicollinearity was detected between independent variables and covariates. Similar procedures without repeated measures were used for the analysis of AUC for all appetite sensations. The SLICE option was used to investigate significant experimental conditions effects in each of the eight predetermined subgroups (based on sex, weight, and restrained eating). The structure of covariance matrix was taken into consideration for all statistical models to ensure an optimal fit to the data. A *p* value < 0.05 was considered statistically significant for all tests, with Bonferroni corrections made for all multiple comparisons. All analyses were performed with Statistical Analysis Software version 9.3 (SAS Institute, Cary, NC, USA).

## 3. Results

### 3.1. Exclusions and Description of Participants

A total of 352 participants were recruited and completed the study. From these participants, 11 (4 men and 7 women) were excluded from the analysis because of missing values in covariates. The remaining total of 341 participants (160 men and 181 women) were thus included in the present study. One missing value was also observed for the initial PFC sensation (time = −10), which led to the exclusion of one participant from the PFC analysis because of the necessity of this covariate in the statistical model. Descriptive characteristics of the participants in each experimental condition are presented in Table 2. No significant differences were found between conditions for any initial characteristic. As explained previously in Section 2.6, all the following results are adjusted for age, time of the day, energy needs, amount eaten, and initial appetite sensations. Note that only initial appetite sensations and total amount eaten contributed significantly to the models.

### 3.2. Objective 1: Effects of Nutrition Claims on Appetite Sensations

No main experimental condition effect was observed for any of the four appetite sensations evaluated, when expressed in both VAS means (*F*_(2,306)_ = 1.14 for desire to eat,* F*_(2,306)_ = 1.54 for hunger,* F*_(2,306)_ = 0.66 for fullness, and* F*_(2,305)_ = 0.68 for PFC; *p* = 1.00) and AUC (*F*_(2,306)_ = 2.99 for desire to eat,* F*_(2,306)_ = 3.20 for hunger,* F*_(2,306)_ = 1.06 for fullness, and* F*_(2,301)_ = 0.4440 for PFC; *p* = 1.00). Mean values for all VAS at each time point (−10, 0, 20, 40, and 60 minutes) as well as their related AUC are presented in Table 3 for each experimental condition.

### 3.3. Objective 2: Effects of Physiological and Psychological Factors on Appetite Sensations

Appetite sensations were affected neither by BMI (*p* = 1.00 for all VAS values and AUC), nor by restrained eating (*p* > 0.96 and *p* > 0.33 for all VAS values and AUC, resp.). However, a main effect of sex was noted for all appetite sensations, with men reporting higher VAS values than women for desire to eat, hunger, and PFC (*p* < 0.004) and lower VAS values than women for fullness (*p* = 0.008). As presented in Table 4, differences were significant at each time point and for all AUCs. Cohen's *d* effect sizes (ESs) ranged from 0.08 to 0.52, which represents small to moderate effects. An effect size comprised between 0.2 and 0.49 represents a small effect size, between 0.5 and 0.79, a moderate effect size, and ≥ 0.8 a large effect size [43]. No sex differences were observed for desire to eat and hunger at baseline, but fullness was higher and PFC lower in women in comparison to men. Note that men had significantly higher energy needs and ate significantly more than women (data not shown). As previously explained in Section 2.6, adjustments were made for these covariates for all results presented.

### 3.4. Objective 3: Combined Effects of Nutrition Claims and Individual Factors on Appetite Sensations

Significant effects of nutrition claims on appetite sensations were observed in specific subgroups (based on combinations of individual factors). Among normal-weight unrestrained women, lower AUC values for desire to eat (ES = 0.76), hunger (ES = 1.03), and PFC (ES = 0.72) were observed in the “diet” condition compared with the “healthy” condition (Table 5). However, significant differences between these two conditions were not observed at each time point. Some differences between “diet” and “hedonic” conditions and also between “healthy” and “hedonic” conditions were also noted among normal-weight unrestrained women. In fact, hunger was lower in the “diet” condition than in the “hedonic” condition at time 0 and was higher in the “healthy” condition than in the “hedonic” condition at time 20 (Table 5). Among overweight/obese unrestrained men, a higher AUC value for fullness was observed in the “diet” condition than in the “healthy” and “hedonic” conditions (ESs of 1.19 and 0.99, resp.) (Table 6). These differences were, however, not observed at each time point. No significant differences were found between other subgroups for any of the four appetite sensations.

## 4. Discussion

The aim of this study was to evaluate the impact of food labeling and individual factors on perceived appetite sensations and more precisely to (1) evaluate the effects of “healthy,” “diet,” or “hedonic” claims on appetite sensations, (2) evaluate if sex, weight, or restrained eating influence appetite sensations, and (3) investigate if sex, weight, and restrained eating can modulate the effects of nutrition claims on appetite sensations. Associated results are that (1) claims did not affect appetite sensations, (2) sex had a significant influence on appetite sensations, but not weight and restrained eating, and (3) a significant interaction between claims and individual factors has been observed in normal-weight unrestrained women and overweight/obese unrestrained men.

As previously reported, the psychological manipulation was effective in changing the “healthy” perception of the snack, as the “healthy” condition was considered to be healthier than the “diet” condition, itself considered healthier than the “hedonic” condition [28]. Contrary to our first hypothesis, the three experimental conditions resulted in similar perceived appetite sensations, suggesting that a snack labeled with healthy characteristics is not necessarily perceived as less satiating than a snack labeled with satiating or hedonic properties. This finding is in contradiction with Finkelstein and Fishbach [18] who demonstrated that participants felt hungrier after eating a “healthy” chocolate-raspberry bar than after eating the same bar described as “yummy.” However, few details are given in this study about the composition of experimental groups, and initial hunger sensation was not measured and then not used as a covariate, as performed in the present study. As our statistical models showed that initial sensations are important covariates to consider in the evaluation of appetite sensations, differences in statistical adjustments between studies may explain the different results obtained. Our finding is also in contradiction with Arguin et al. [20] who demonstrated that highlighting the satiating properties of a meal increases satiety potential against a control condition. The different types of food (i.e., snack versus meal) used and the absence of a control condition (i.e., without any description) in our study may explain why we did not observe such a difference. It could also be explained by the fact that our “diet” condition was not only targeting satiating properties, but also weight control. As we did not measure the expected satiating power (as performed previously for perceived healthiness) [28], we cannot distinguish between expected and perceived appetite sensations. In any case, the absence of an effect of nutrition claims on appetite sensations in our study implies neither a contraindication nor incentive to their use in a public health context.

As hypothesized, sex had a significant influence on the perception of appetite sensations, regardless of nutrition claims, energy needs, and amount eaten (men had significantly higher energy needs and ate significantly more than women). Indeed, men reported higher levels of desire to eat, hunger, and PFC and lower levels of fullness. These results suggest that women could feel more easily satiated than men and could be less tempted to prospectively eat more food. Some studies have already reported similar findings [7, 44], while other studies found no sex differences [8] or mixed results depending on the appetite sensation observed and experimental conditions (*ad libitum* versus controlled) [46]. The fact that baseline fullness and PFC were, respectively, higher and lower in women than in men is also of interest. It suggests that these two sensations could differ between men and women even before food consumption. Sex differences in the perception of appetite sensations could be explained by differences in hormonal profiles [47]. Men and women could also differ in their neurological response to satiation [48]. These observations involve that public health nutrition interventions may not have the same effects in men and women and that individual or group interventions should be adapted in consequence. According to our findings, women could be more responsive to an intuitive or mindful eating approach than men, that is, to eat in response to physical sensations such as hunger and satiety signals [49–51]. Health at Every Size® (HAES) programs, endorsed by the Association for Size Diversity and Health, are an example of interventions addressing the recognition of appetite sensations. “*Choisir de maigrir?”* (“What about losing weight?”) is an example of HAES program developed specifically for women [52]. Other programs are also sex-specific, focusing more on body image for women and energy and body functions for men [53].

Contrary to our hypothesis, BMI did not significantly influence appetite sensations. Current literature shows that obese individuals have lower fasting ghrelin levels and that their ghrelin response to food intake is less pronounced than in normal-weight individuals [54]. This is in line with the proposition that overweight or obese individuals could be less sensitive to their internal appetite sensations [26]. However, we found no difference between normal-weight and overweight/obese individuals, which has been found in past literature [44]. Another study proposes that hormonal response to food intake could be dependent of a BMI by sex interaction [47], suggesting that the study of BMI alone is not sufficient. Appetite-regulating hormones are not necessarily related to subjective appetite sensations [55], which could explain why we obtained different results as we only measured perceived appetite sensations and not appetite-regulating hormones. Also, we did not differentiate between overweight and obese individuals, our sample including more overweight (*n* = 124) than obese (*n* = 51) individuals.

No main effect of restraint status on appetite sensations was observed. This was unexpected as restrained eating has been previously associated with higher levels of perceived hunger [56]. It has also been already associated with higher levels of ghrelin [24, 57]. Hooper et al. [58] also demonstrated that a high degree of weight cycling was associated with an appetite-stimulating hormonal profile. As mentioned previously, however, appetite-regulating hormones are not necessarily well related to subjective appetite sensations. On the other hand, other studies found no effect of restrained eating on hunger [44, 55]. Restrained eating can be measured either by the Restraint Scale (as performed in the present study) or by the restraint scales included in the Three-Factor Eating Questionnaire (TFEQ) [59] or the Dutch Eating Behavior Questionnaire (DEBQ) [60]. The Restraint Scale combines the measure of concern with dieting and weight fluctuation, whereas both TFEQ and DEBQ measure attempt to restrict eating, independent of weight fluctuation. The choice of the questionnaire used to classify participants as restrained or not could then explain differences in results obtained between studies. As proposed earlier, it is also possible that restrained eating needs to be considered in interaction with sex or BMI to reveal significant effects. For example, Martins et al. [61] showed that restrained eating in women could be associated with lower levels of hunger and higher levels of fullness. Even if weight status and restrained eating are important factors to consider in individual consultation, our results suggest that they do not necessarily need to be taken into account for the evaluation of appetite sensations.

To our knowledge, this is the first study to explore the influence of nutrition claims on appetite sensations according to a combination of individual factors, namely, sex, weight, and restrained eating. Our hypothesis that overweight/obese restrained women would be more easily satiated in the “diet” and “hedonic” conditions in comparison with the “healthy” condition was not confirmed as a satiating effect of the “diet” condition was rather observed in normal-weight unrestrained women and overweight/obese unrestrained men, while no satiating effect of the “hedonic” condition was noted. Being more easily influenced by satiety-related claims could either indicate a greater susceptibility to external factors, or in contrary a better predisposition for the recognition of internal satiety signals. An effect of nutrition claims was only noticed in individuals presenting a low level of cognitive restraint, which has already been associated with a lower susceptibility to external influences [20, 56]. It is then more likely that the “diet” claim led to an awareness of internal sensations. This observation highlights the relevance of considering individual factors in interaction when studying appetite sensations. Our results also suggest that nutrition claims emphasizing satiating properties could be more effective in reducing perceived hunger than claims focusing on hedonic properties. More precisely, it suggests a potential benefit of satiety-related claims, at least in certain subgroups of individuals.

This study has some limitations. First, it might be argued that the “healthy” and “diet” labels were not congruent with the food tasted, which could explain the absence of a main effect on appetite sensations. However, this argument is not supported as the manipulation effectively changed the perception of food [28]. On the other hand, no control condition (i.e., without any description) was used, and the “diet” condition was not only targeting satiating properties but also weight management. Also, the amount eaten was not standardized, which however allowed participants to eat according to their physiological and psychological needs. The use of many multiple comparisons and Bonferroni corrections could have led to a lack of power, but it can also be argued that the probability of committing type 1 errors is really small. This study also has strengths, such as a high number of participants recruited on the basis of a factorial design. This is also the first study to evaluate the role played by specific combinations of individual factors in the study of the effect of nutrition claims on appetite sensations.

## 5. Conclusions

We investigated the impact of nutrition claims and individual factors on the perception of appetite sensations. Women felt more satiated then men regardless of nutrition claims, while BMI and restrained eating alone did not influence appetite sensations. No adverse effect of nutrition claims on appetite sensations was observed, which suggests no contraindication to their use. Positive effects of satiety-related claims have been observed only in specific subgroups of individual. In a real-world setting, foods labeled with satiety-related claims would possess demonstrated physiological satiating properties and therefore would probably induce a physiological effect (as opposed to a psychological effect in this study). As enhanced satiety may benefit dietary control or weight-management [9], it may thus be useful to inform consumers about these specific properties.

To conclude, nutrition claims do not generally affect appetite sensations and the evaluation of their relevance should rather be based on eating or buying behavior variables. Sex is an important individual factor to consider when studying appetite sensations, while weight and restrained eating should be considered in interaction for a better understanding of their effects. More studies are needed to assess a significant and beneficial effect of nutrition claims on appetite sensations and eating behavior, and to better understand the contribution of individual factors.

## Figures and Tables

**Table 1:** Factorial distribution of participants (*n*).

	Men (*n* = 160^*∗*^)				Women (*n* = 181^*∗*^)			
	Normal-weight (*n* = 71)		Overweight/obese (*n* = 89)		Normal-weight (*n* = 95)		Overweight/obese (*n* = 86)	
	Unrestrained (*n* = 43)	Restrained (*n* = 28)	Unrestrained (*n* = 37)	Restrained (*n* = 52)	Unrestrained (*n* = 54)	Restrained (*n* = 41)	Unrestrained (*n* = 37)	Restrained (*n* = 49)
Healthy	13	9	13	18	17	14	11	21
Diet	18	10	11	16	20	11	13	13
Hedonic	12	9	13	18	17	16	13	15

^*∗*^Numbers after exclusions from analysis.

**Table 2:** Characteristics of participants for the total sample and each experimental condition.

	Total (*n* = 341)	Healthy (*n* = 116)	Diet (*n* = 112)	Hedonic (*n* = 113)	*p* values
Men	160 (47)^*∗*^	53 (46)	55 (49)	52 (46)	
Women	181 (53)	63 (54)	57 (51)	61 (54)	
Age (y)	37.8 ± 15.0	38.3 ± 15.3	37 ± 15.2	38.0 ± 14.7	0.8055
BMI (kg/m^2^)	25.5 ± 4.4	25.9 ± 4.8	25.1 ± 4.3	25.5 ± 4.1	0.3617
Normal weight	166 (49)	53 (46)	59 (53)	54 (48)	
Overweight/obese	175 (51)	63 (54)	53 (47)	59 (52)	
Restraint score	13.1 ± 4.7	13.1 ± 4.7	12.9 ± 4.8	13.3 ± 4.7	0.8250
Unrestrained	171 (50)	54 (47)	62 (55)	55 (49)	
Restrained	170 (50)	62 (53)	50 (45)	58 (51)	
Energy needs (kcal)	2041 ± 505	2101 ± 666	2037 ± 432	1984 ± 357	0.2131
Time of the day (h)	12:40 ± 2:35	12:35 ± 2:40	12:40 ± 2:40	12:50 ± 2:30	0.8793
Appreciation of the food (mm)	108.9 ± 19.3	112.0 ± 18.3	107.9 ± 18.4	106.5 ± 19.3	0.8214
FPS score	49.6 ± 7.4	49.6 ± 7.5	50.1 ± 7.3	49.1 ± 7.4	0.5704

^*∗*^Values are expressed as mean ± standard deviation or number (percentage).

**Table 3:** Appetite VAS values (mm) at each time point and their related AUC (mm × 60 min) for each experimental condition.

	Time	Healthy	Diet	Hedonic	Unadjusted *p* values	Adjusted *p* values^a^
Desire to eat	−10 min	76.9^*∗*^ ± 37.7	83.8 ± 35.9	84.6 ± 30.0	0.1861	0.5583
	0 min	46.2 ± 32.0	47.6 ± 34.8	52.3 ± 35.2	0.8354	1.0000
	20 min	42.3 ± 31.7	41.1 ± 31.9	46.1 ± 33.2	0.4857	1.0000
	40 min	46.3 ± 35.7	42.5 ± 34.0	50.2 ± 35.5	0.2282	0.6846
	60 min	53.7 ± 37.7	47.2 ± 34.8	53.9 ± 38.2	0.1081	0.3243
	AUC	2772 ± 1852	2619 ± 1857	2989 ± 1864	1.0000	1.0000

Hunger	−10 min	74.8 ± 39.9	77.7 ± 35.9	75.5 ± 34.6	0.8224	1.0000
	0 min	39.1 ± 31.4	38.1 ± 31.6	43.4 ± 34.9	0.4469	1.0000
	20 min	40.0 ± 32.0	38.3 ± 31.6	41.8 ± 32.9	0.3979	1.0000
	40 min	44.0 ± 34.0	41.7 ± 33.0	49.5 ± 35.5	0.0893	0.2679
	60 min	52.1 ± 37.2	48.0 ± 35.6	52.7 ± 38.5	0.3669	1.0000
	AUC	2592 ± 1836	2461 ± 1829	2788 ± 1884	1.0000	1.0000

Fullness	−10 min	48.3 ± 34.6	46.2 ± 33.1	49.8 ± 32.4	0.7143	1.0000
	0 min	86.8 ± 33.9	88.9 ± 36.0	84.1 ± 34.8	0.4331	1.0000
	20 min	82.2 ± 36.1	85.6 ± 36.9	82.7 ± 34.9	0.6704	1.0000
	40 min	75.0 ± 38.3	81.2 ± 36.2	77.5 ± 36.7	0.2133	0.6399
	60 min	71.1 ± 38.2	75.1 ± 39.2	76.1 ± 38.1	0.6941	1.0000
	AUC	4725 ± 2000	4974 ± 2034	4806 ± 1932	1.0000	1.0000

PFC	−10 min	78.8 ± 29.1	79.3 ± 31.4	82.0 ± 27.2	0.6797	1.0000
	0 min^b^	49.2 ± 31.2	48.3 ± 32.7	53.3 ± 33.2	0.5588	1.0000
	20 min^c^	48.1 ± 32.5	47.5 ± 32.8	52.7 ± 33.9	0.3482	1.0000
	40 min^d^	54.7 ± 34.2	52.4 ± 33.2	57.0 ± 34.4	0.6696	1.0000
	60 min^e^	62.5 ± 38.0	57.9 ± 34.4	64.3 ± 35.1	0.5808	1.0000
	AUC^f^	3175 ± 1874	3053 ± 1867	3384 ± 1814	1.0000	1.0000

^*∗*^Values are expressed as mean ± standard deviation. ^a^Bonferroni adjustments. ^b^Missing value (*n* = 1; diet: 1). ^c^Missing values (*n* = 2; hedonic: 2). ^d^Missing value (*n* = 1; hedonic: 1). ^e^Missing values (*n* = 4; diet: 1; hedonic: 3). ^f^Missing values (*n* = 4; diet: 1; hedonic: 3).

**Table 4:** VAS values (mm) at each time point and their related and AUC (mm × 60 min) in men and women.

		Men	Women	*p* values
Desire to eat	−10 min	82.0 ± 32.7	81.5 ± 36.6	0.8837
	0 min	50.2 ± 33.3	47.3 ± 34.6	**0.0014**
	20 min	48.9 ± 32.6	38.1 ± 31.2	**<0.0001**
	40 min	51.4 ± 33.8	41.9 ± 35.7	**<0.0001**
	60 min	57.3 ± 34.6	46.7 ± 38.3	**<0.0001**
	AUC	3081 ± 1849	2540 ± 1834	**<0.0001**

Hunger	−10 min	77.3 ± 34.7	74.9 ± 38.6	0.5408
	0 min	42.8 ± 32.1	37.9 ± 33.0	**0.0007**
	20 min	45.9 ± 32.8	34.9 ± 30.7	**<0.0001**
	40 min	51.3 ± 34.3	39.5 ± 33.4	**<0.0001**
	60 min	57.9 ± 35.1	44.8 ± 37.8	**<0.0001**
	AUC	2953 ± 1870	2315 ± 1783	**<0.0001**

Fullness	−10 min	43.3 ± 31.2	52.3 ± 34.6	**0.0122**
	0 min	84.2 ± 33.9	88.7 ± 35.6	**0.0208**
	20 min	77.4 ± 34.7	88.9 ± 36.1	**<0.0001**
	40 min	71.3 ± 35.9	83.7 ± 37.3	**<0.0001**
	60 min	70.7 ± 35.2	77.1 ± 40.9	**0.0068**
	AUC	4522 ± 1953	5109 ± 1979	**<0.0001**

PFC	−10 min	85.8 ± 27.6	74.8 ± 29.8	**0.0005**
	0 min^a^	56.3 ± 32.4	44.9 ± 31.4	**<0.0001**
	20 min^b^	57.7 ± 33.0	42.0 ± 31.4	**<0.0001**
	40 min^c^	63.6 ± 33.0	46.8 ± 32.7	**<0.0001**
	60 min^d^	69.7 ± 33.6	54.2 ± 36.5	**<0.0001**
	AUC^e^	3697 ± 1848	2759 ± 1745	**<0.0001**

Values are expressed as mean ± standard deviation. ^a^Missing value (*n* = 1; woman: 1). ^b^Missing values (*n* = 2; women: 2). ^c^Missing value (*n* = 1; woman: 1). ^d^Missing values (*n* = 4; man: 1; women: 3). ^e^Missing values (*n* = 4; man: 1; women: 3).

**Table 5:** Desire to eat, hunger, and PFC in normal-weight unrestrained women for each experimental condition.

		Healthy	Diet	Hedonic	Unadjusted *p* values	Adjusted *p* values^c^
Desire to eat	−10 min	76 ± 42	84 ± 40	91 ± 31	0.5487	1.0000
	0 min	51 ± 34	38 ± 29	61 ± 42	0.0904	0.2712
	20 min	46 ± 37^a^	25 ± 23^b^	32 ± 34^a,b^	0.0204	0.0612
	40 min	54 ± 38^a^	28 ± 26^b^	37 ± 43^a,b^	0.0049	**0.0147**
	60 min	61 ± 40^a^	35 ± 28^b^	45 ± 43^a,b^	0.0130	**0.0390**
	AUC	3126 ± 2089^a^	1785 ± 1374^b^	2446 ± 1948^a,b^	0.0019	**0.0057**

Hunger	−10 min	74 ± 45	68 ± 35	85 ± 38	0.4357	1.0000
	0 min	47 ± 35^a^	22 ± 21^b^	48 ± 42^a^	0.0047	**0.0141**
	20 min	49 ± 37^a^	20 ± 19^b^	30 ± 33^b^	0.0046	**0.0138**
	40 min	54 ± 36^a^	23 ± 19^b^	36 ± 41^a,b^	0.0028	**0.0084**
	60 min	59 ± 38^a^	31 ± 28^b^	44 ± 42^a,b^	0.0279	0.0837
	AUC	3114 ± 2100^a^	1402 ± 1047^b^	2243 ± 1998^a,b^	0.0031	**0.0093**

PFC	−10 min	70 ± 30	66 ± 32	75 ± 33	0.7139	1.0000
	0 min	50 ± 30	32 ± 21	51 ± 38	0.0585	0.1755
	20 min	50 ± 34	30 ± 22	26 ± 24	0.0310	0.0930
	40 min	55 ± 36	34 ± 22	31 ± 24	0.0391	0.1173
	60 min	64 ± 42	45 ± 28	43 ± 31	0.1469	0.4407
	AUC	3233 ± 1974^a^	2048 ± 1233^b^	2063 ± 1264^a,b^	0.0207	0.0621

^a,b^Values on the same line with different superscript letters are significantly different (*p* < 0.05). ^c^Least squares means with bonferroni adjustement for multiple comparisons.

**Table 6:** Fullness in overweight/obese unrestrained men for each experimental condition.

		Healthy	Diet	Hedonic	Unadjusted *p* values	Adjusted *p* values^c^
Fullness	−10 min	41 ± 30	61 ± 37	53 ± 18	0.2460	1.0000
	0 min	68 ± 33	99 ± 36	75 ± 22	0.1076	0.3228
	20 min	63 ± 34	98 ± 35	73 ± 29	0.0666	0.1998
	40 min	56 ± 35^a,b^	100 ± 34^a^	63 ± 34^b^	0.0036	**0.0108**
	60 min	55 ± 27	90 ± 38	65 ± 31	0.0525	0.1575
	AUC	3607 ± 1745^b^	5847 ± 2008^a^	4104 ± 1469^b^	0.0012	**0.0036**

^a,b^Values on the same line with different superscript letters are significantly different (*p* < 0.05). ^c^Least squares means with bonferroni adjustement for multiple comparisons.

## Abbreviations

BMI: Body Mass Index
DEBQ: Dutch Eating Behavior Questionnaire
ES: Effect size
FFQ: Food Frequency Questionnaire
FPS: Food Pleasure Scale
HAES: Health at Every Size
PFC: Prospective food consumption
TFEQ: Three-Factor Eating Questionnaire
VAS: Visual analog scale.
